# Mitochondrial Topoisomerase I is Critical for Mitochondrial Integrity and Cellular Energy Metabolism

**DOI:** 10.1371/journal.pone.0041094

**Published:** 2012-07-20

**Authors:** Céline Douarre, Carole Sourbier, Ilaria Dalla Rosa, Benu Brata Das, Christophe E. Redon, Hongliang Zhang, Len Neckers, Yves Pommier

**Affiliations:** 1 Laboratory of Molecular Pharmacology, National Cancer Institute, National Institutes of Health, Bethesda, Maryland, United States of America; 2 Urologic Oncology Branch, National Cancer Institute, National Institutes of Health, Bethesda, Maryland, United States of America; University of Medicine and Dentistry of New Jersey, United States of America

## Abstract

**Background:**

Mitochondria contain their own DNA genome (mtDNA), as well as specific DNA replication and protein synthesis machineries. Relaxation of the circular, double-stranded mtDNA relies on the presence of topoisomerase activity. Three different topoisomerases have been identified in mitochondria: Top1mt, Top3α and a truncated form of Top2β.

**Methodology/Principal Findings:**

The present study shows the importance of Top1mt in mitochondrial homeostasis. Here we show that Top1mt−/− murine embryonic fibroblasts (MEF) exhibit dysfunctional mitochondrial respiration, which leads decreased ATP production and compensation by increased glycolysis and fatty acid oxidation. ROS production in Top1mt−/− MEF cells is involved in nuclear DNA damage and induction of autophagy. Lack of Top1mt also triggers oxidative stress and DNA damage associated with lipid peroxidation and mitophagy in Top1mt−/− mice.

**Conclusion/Significance:**

Together, our data implicate Top1mt for mitochondrial integrity and energy metabolism. The compensation mechanism described here contributes to the survival of Top1mt−/− cells and mice despite alterations of mitochondrial functions and metabolism. Therefore, this study supports a novel model for cellular adaptation to mitochondrial damage.

## Introduction

In mammalian cells, mitochondrial DNA (mtDNA) represents a significant fraction of the genetic material (up to 1% in some tissues). Each mitochondrion contains 2 to10 mtDNA genomes. Each circular, double-stranded mtDNA genome encodes 13 core proteins that are part of the mitochondrial electron chain responsible for oxidative phosphorylation. It also codes for 22 transfer RNAs and the 12S and 16S ribosomal RNAs [1]. One of the most important mitochondrial functions is to generate approximately 90% of cellular ATP through oxidative phosphorylation (OXPHOS), involving chemical reactions that link the oxidation of NADH and FADH_2_ to the phosphorylation of ADP. The rest of ATP production is provided by glycolysis [2].

DNA Topoisomerases are ubiquitous enzymes that control and adjust the topologic states of DNA [3], [4]. They catalyze the transient cleavage and rejoining of DNA, which allows DNA strands to move around each other, thereby relieving the torsional stress introduced in DNA during replication and transcription. Three topoisomerases, Top1mt [5], Top3α [6] and Top2β [7] have been found in mitochondria. Mitochondrial DNA topoisomerase I (Top1mt) is the only mitochondrial topoisomerase encoded by a specific gene for mitochondria. It is present in all vertebrates [5], [8], implicating a functional role of Top1mt in mtDNA maintenance and topology [9].

Mitochondrial dysfunction causes a decrease in ATP production, oxidative damage and induction of apoptosis, all of which are involved in the pathogenesis of a growing number of neurological, muscular and metabolic disorders [10], [11]. Cells defective for mitochondrial respiration generate their energy from an enhancement of glycolysis, described as the Warburg effect in cancer cells [12], and which leads to a shift in metabolism away from aerobic respiration toward glycolysis, even when sufficient oxygen is present to support respiration. Accumulation of mtDNA damage has been reported in neurodegenerative disorders (Parkinson, Alzheimer and Huntington diseases), myopathies and diabetes, and associated with cancer, aging and other age-related degenerative disorders [11], [13], [14].

Although mitochondria have their own genome, most of the mitochondrial proteins and all the enzymes required for mtDNA homeostasis are encoded in the nuclear genome [15] including Top1mt [5]. Moreover, mitochondrial functions are regulated by a wide range of transcription factors encoded by the nucleus [16], including mitochondrial transcription factors A (TFAM) and B (TFB1M, TFB2M), nuclear respiratory factor 1 (NRF-1), GA binding proteins (GABPα, GABPβ2), peroxisome proliferator–activated receptors (PPAR-α, PPAR-γ), PPAR-γ coactivators (PGC-1α, PPAR-β) and c-myc [17], [18].

Mitochondrial biogenesis and function are dynamically regulated in tissue- and signal-specific manners to enable cellular adaptation to energetic and metabolic demands. Coordination between expression of the nuclear and mitochondrial genomes is an essential feature of eukaryotic cells. For example, mitochondrial-to-nuclear signaling, which is referred to as retrograde regulation, regulates the nuclear genome to change mitochondrial genome or function [19], [20]. These changes involve responses to ROS (reactive oxygen species) and free radicals generated from respiratory chain [16]. Abnormally high levels of ROS production contribute to oxidative damage, aging, cancers and cell death. However, ROS can also trigger the stimulation of mitochondrial proliferation to supply energy for cell survival, repair of cellular damages and synthesis of new proteins [16], [21]. Mitochondrial biogenesis occurs also in response to DNA damage [22], [23]. DNA damage induced by DNA topoisomerase II-inhibitors (etoposide, mitoxantrone) and ionizing radiation [24] have been shown to induce up-regulation of mitochondrial biogenesis.

In the present study, we analyzed the cellular impacts of Top1mt deficiency using Top1mt knockout (Top1mt−/−) MEF cells and mice. Biochemical analyses indicate that Top1mt deficiency results in mitochondrial dysfunctions with enhancement of glycolysis, induction of the DNA damage response (DDR) pathways, activation of autophagy, fatty acid oxidation and lipid peroxidation. Absence of Top1mt in mice triggers mitophagy in liver, oxidative stress and lipid peroxidation. This is the first report demonstrating mitochondrial dysfunction in Top1mt-deficient cells and the involvement of Top1mt in the maintenance of cellular homeostasis.

## Results

### Mitochondria are Dysfunctional in Top1mt−/− Cells

**Figure 1:**
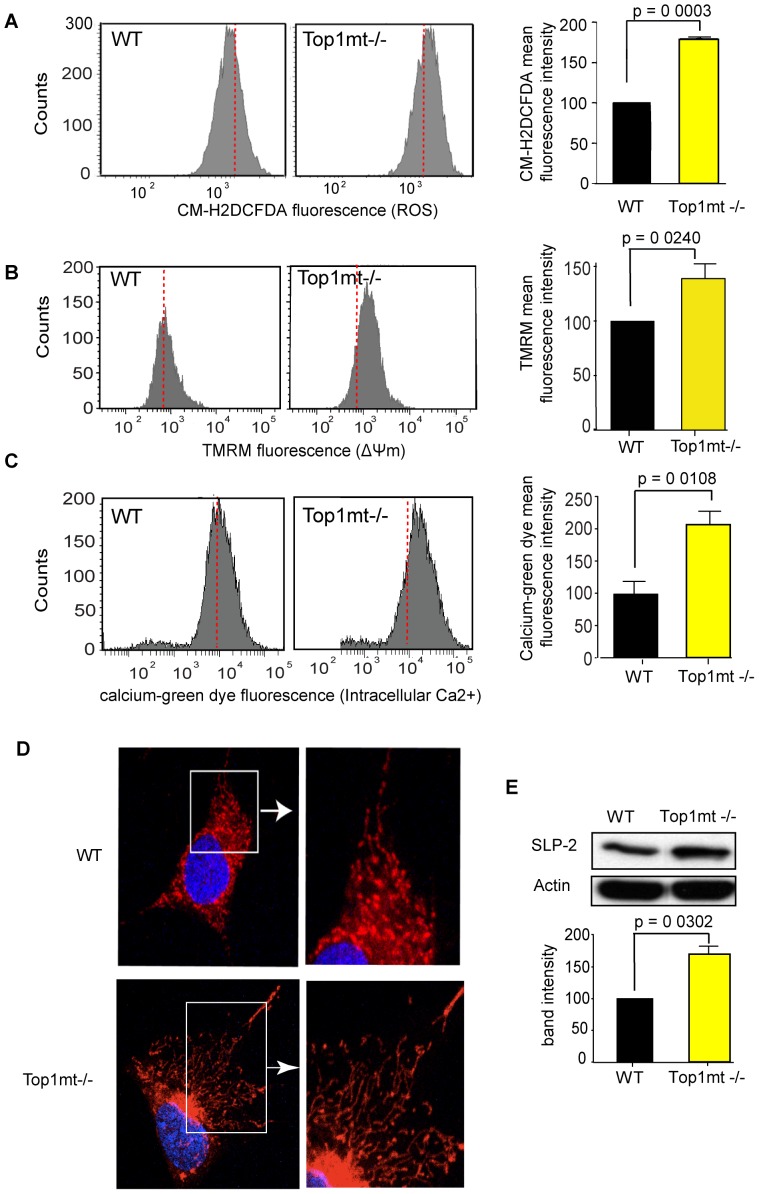
Mitochondrial dysfunction in Top1mt−/− cells. FACS analysis of ROS (**A**), mitochondrial membrane potential (ΔΨm) (**B**) and intracellular calcium content (**C**) in WT and Top1mt−/− cells. CM-H_2_DCFDA, TMRM and Calcium Green dye fluorescence were plotted against cells numbers (count). Red line represents median of the histogram for WT samples. For each panel, quantification of mean fluorescence intensity is shown on the right referred as percentage of WT values. (**D**) Mitochondrial hyperfusion in Top1mt−/− cells visualized by mitotracker red staining. (**E**) SLP-2 (Stomatin like protein 2) expression by Western-blot (cropped figure) in WT and Top1mt−/− cells. Right plots represent means ± standard deviations of at least 3 experiments (quantification from 3 independent determinations).

To assess mitochondrial dysfunction in Top1mt −/− cells, we measured ROS production using the CM-H_2_DCFDA cell-permeant indicator. Figure 1A shows enhanced ROS production in Top1mt−/− cells. We next checked mitochondrial membrane potential by treating cells with the lipophilic cation TMRM, which accumulates in active mitochondria in a membrane potential-dependent manner and emits a bright red-orange fluorescence. The increase of TMRM fluorescence signal (Fig. 1B) indicates hyperpolarization of mitochondrial membranes in Top1mt−/− cells. Because mitochondrial dysfunctions are generally associated with an increase of cytoplasmic Ca^2+^ [25], we also assessed intracellular calcium contents in Top1mt−/− cells using a FACS assay with a calcium-green dye. Our data (Fig. 1C) show an increase of intracellular calcium concentration in Top1mt−/− cells. We next checked mitochondrial morphology with Mitotracker Red staining. Figure 1D shows a representative image demonstrating the presence of hyperfused mitochondria, as shown by the presence of highly interconnected network in Top1mt−/− cells. Mitochondrial hyperfusion is usually found in MEF cells after stress induction [26], and is mediated by SLP-2 protein (Stomatin-like protein 2) [26]. Because it has been shown that SLP-2 protein levels increase in HeLa cells under mitochondrial stress [27], we studied SLP-2 expression in Top1mt−/− and WT cells. Figure 1E shows that SLP-2 is increased by ∼60% in Top1mt−/− cells. Taken together, our results suggest the presence of dysfunctional mitochondria in Top1mt−/− cells.

### The Retrograde Response is Activated in Top1mt −/− Cells

**Figure 2:**
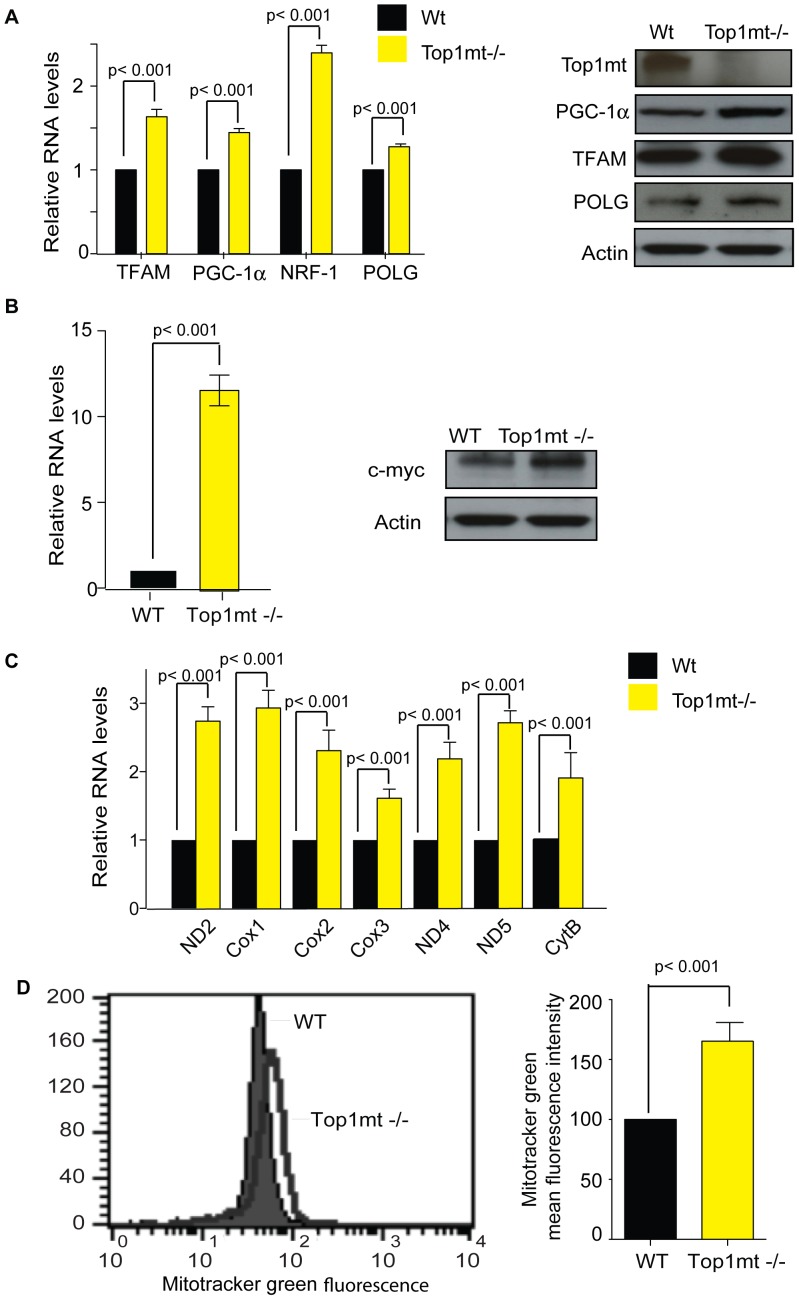
Induction of the retrograde response in Top1mt−/− cells. (**A**) Gene expression of nuclear-encoded TFAM, PGC-1α, NRF-1 and POLG by RT-PCR in WT and Top1mt−/− cells (means ± standard deviations of 3 experiments). Gene expression was normalized to β2-microglobulin (β2M). Right panels show representative Western blots for Top1mt, PGC-1α, TFAM, POLG and actin protein levels in WT and Top1mt−/− cells. (**B**) Myc gene expression by RT-PCR normalized to β2M (mean ± standard deviation of 3 experiments) and c-myc protein level by Western blotting in WT and Top1mt−/− cells. (**C**) ND2, Cox1, Cox2; Cox3; ND4; ND5 and CytB expression by RT-PCR in WT and Top1mt−/− cells (means ± standard deviations of 3 experiments). (**D**) Determination of mitochondrial mass by Mitotracker Green staining. Representative histogram corresponding to Mitotracker Green fluorescence (x axis) plotted against cells numbers (count) and quantification of Mitotracker Green mean fluorescence intensity in cell population referred as percentage of WT values.

Because oxidative stress and mitochondrial dysfunction tend to induce a compensatory biogenesis of mitochondria by activation of nuclear genes involved in mitochondrial biogenesis [16], [22], [28], we analyzed the expression of mitochondrial genes and nuclear-encoded genes that activate mitochondria in Top1mt−/− and WT cells. As shown in Figure 2A, Top1mt−/− cells show increased mRNA expression for TFAM, a regulator of mtDNA replication and transcription, for PGC-1, the master regulator of mitochondrial biogenesis, for NRF-1, a transcription factor for nuclear-encoded mitochondrial genes [29], [30], and for POLG, the sole DNA polymerase responsible for replication of the mitochondrial genome [2]. PGC-1, POLG, TFAM and protein overexpression in Top1mt −/− cells were confirmed by Western blotting (Fig. 2A).

We recently reported that c-myc is a key regulator for nuclear-encoded mitochondrial genes including TOP1mt [31]. c-myc also promotes mitochondrial biogenesis by upregulating TFAM [17]. In this context, we looked at c-myc transcription and protein expression in Top1mt−/− and WT cells. Figure 2B shows that c-myc mRNA and protein are overexpressed in Top1mt −/− cells.

We then analyzed mtDNA transcription in Top1mt−/− cells. Figure 2C shows increased expression of Cox1, Cox2, Cox3, ND2, ND4, ND5 and CytB in cells lacking Top1mt compared to WT cells. To validate whether the increase of mitochondrial and nuclear-encoded genes involved in mitochondrial biogenesis is associated with increase of mitochondrial mass, we performed a Mitotracker Green staining in WT and Top1mt−/− cells. Mitochondrial mass was more abundant in Top1mt−/− cells (166%) compared to WT cells (100%). Taken together these data show activation of the retrograde response in Top1mt−/− cells.

### Enhanced Glycolysis in Top1mt −/− Cells

**Figure 3:**
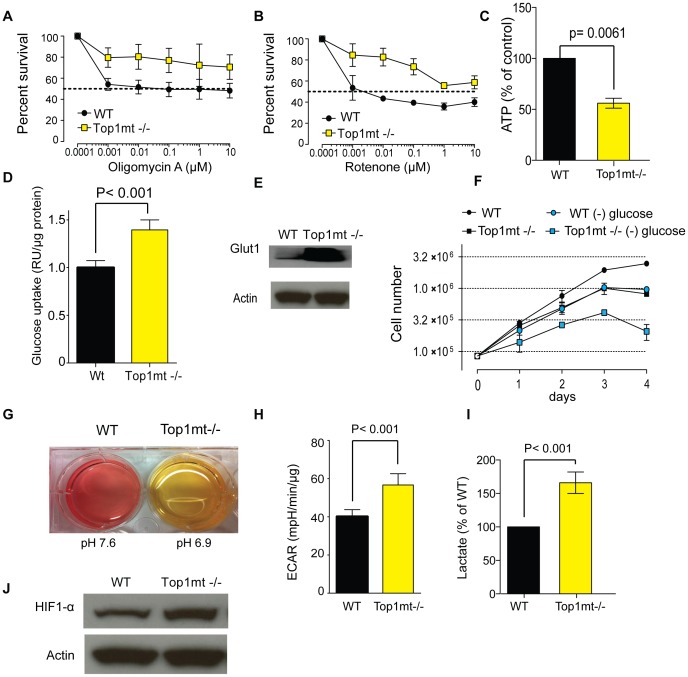
Top1mt deficiency leads to increased glycolytic activity. Cytotoxicity effect of (**A**) oligomycin A and (**B**) rotenone, measured by MTS assay for 72 h in WT and Top1mt−/− cells (means ± standard deviations of 3 experiments). (**C**) ATP content in WT and Top1mt−/− cells (mean ± standard deviation of 3 experiments). (**D**) Differential glucose uptake in WT and Top1mt−/− cells measured with fluorescent 2-NBDG (means ± standard deviations of 3 experiments). (**E**) Glut1 (glucose transporter 1) expression by Western blotting in WT and Top1mt−/− cells. (**F**) Proliferation test by trypan blue exclusion and survival rate of WT (circles) and Top1mt−/− cells (squares) in the presence (black) or absence (blue) of glucose (means ± standard deviations of 2 experiments. (**G**) Representative experiment showing pH determination after 8 days of culture in WT and Top1mt−/− cells. (**H**) Extra-cellular acidification rate (ECAR) determined by Seahorse analysis in WT and Top1mt−/− cells (means ± standard deviations of 3 experiments). (**I**) Lactate content in WT and Top1mt−/− cells (mean ± standard deviation of 3 experiments). (**J**) Representative experiment showing HIF1-α protein expression by Western blotting in WT and Top1mt−/− cells.

To assess the functionality of the respiratory chain in Top1mt−/− cells, we next treated in parallel Top1mt−/− and WT cells with rotenone and oligomycin A, two specific inhibitors of the respiratory chain, [32]. Rotenone inhibits the transfer of electrons from iron-sulfur centers in complex I to ubiquinone. Oligomycin A blocks the proton channel (Fo subunit) of ATP synthase, which is necessary for oxidative phosphorylation of ADP to ATP. Survival assays show that Top1mt−/− cells are resistant to oligomycin A (Fig. 3A) and rotenone (Fig3. B), suggesting that Top1mt−/− cells are less dependent on the electron transport chain for ATP production than WT cells. Subsequently, we checked ATP in Top1mt−/− cells. Not surprisingly, the ATP content was 2-fold less in Top1mt−/− compared to WT cells (Fig. 3C), confirming dysfunctional respiratory chain in Top1mt−/− cells.

Because glycolysis is an alternative for mitochondrial ATP production in cells, we next tested glucose uptake in Top1mt−/− cells. Our data indicate a 50% higher glucose uptake in Top1mt−/− (Fig. 3D). In agreement with these results, glucose transporter 1 (Glut1) was strongly overexpressed in cells lacking Top1mt (Fig. 3E). Moreover, glucose starvation leads to 45% and 60% growth reduction in WT and Top1mt−/− cells respectively after 3 days, and 60% and 75% after 4 days. Thus, the proliferation of Top1mt−/− cells tended to be selectively impaired by glucose starvation, compared to WT cells (Fig. 3F), consistent with the importance of glucose consumption for Top1mt−/− cells proliferation.

Another noticeable characteristic of the Top1mt−/− cells is an acidification of the culture medium upon continuous cell culture. After 8 days without addition of fresh medium, the pH of the Top1mt−/− cell cultures dropped to 6.9 vs. 7.6 for the WT cells (Fig. 3G). As acidification of the medium is one of the features associated with enhanced glycolysis, we measured extracellular acidification rate in Top1mt−/− and WT cells, and found an increase in the extra-cellular acidification rate (ECAR) in Top1mt−/− cells (Fig. 3H). The ECAR increase was associated with a corresponding increased lactate production (Fig. 3I). Because HIF1-α protein (Hypoxia-inducible factor 1) is known to activate the transcription of genes involved in glucose transport and glycolysis [33], [34], we measured its expression. Figure 3J shows that HIF1-α is upregulated in Top1mt−/− cells, reinforcing the findings that glycolysis is enhanced in cells lacking Top1mt.

### Enhanced Fatty Acid Oxidation and Oxidative Damage in Top1mt −/− Cells and Mice

**Figure 4:**
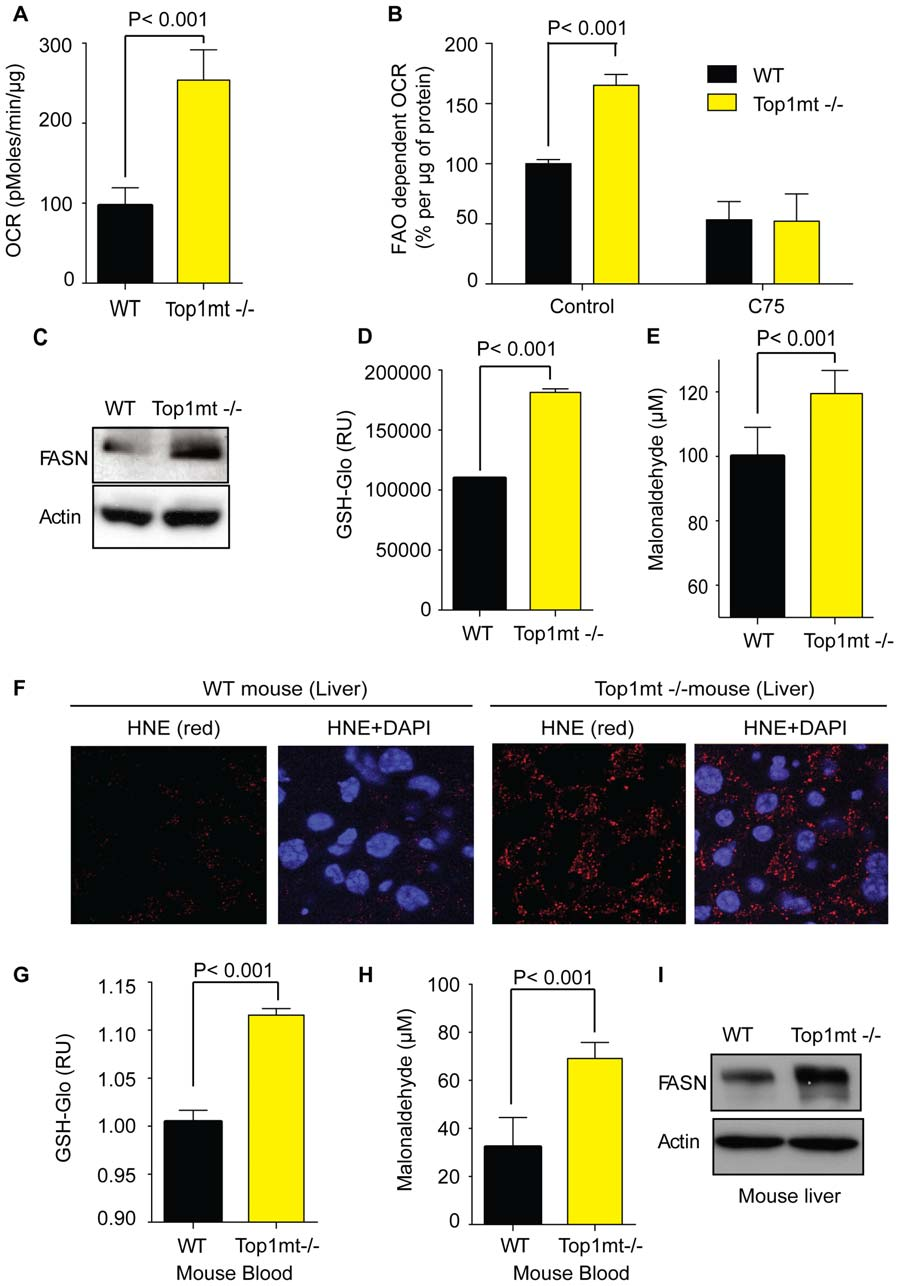
Increase of fatty acid oxidation and lipogenesis in Top1mt-deficient cell line and Top1mt−/− mouse liver. (**A**) Oxygen consumption rate (OCR) in WT and Top1mt−/− cells (means ± standard deviations of 3 experiments). (**B**) Fatty acid oxidation dependent oxygen consumption rate in WT and Top1mt−/− cells after a 10 µg/ml C75 treatment for 3 h (means ± standard deviations of 3 experiments) (**C**) Representative experiment showing FASN (Fatty Acid Synthase) expression by Western blotting in WT and Top1mt−/− cells. (**D**) Glutathione concentration in WT and Top1mt−/− cells (means ± standard deviations of 3 experiments). (**E**) Malonaldehyde (MDA) measurement in WT and Top1mt −/− cells. (**F**) HNE (4-Hydroxynonenal) by immunofluorescence staining in control and Top1mt −/− mice liver. (**G**) Glutathione concentration in blood of control and Top1mt−/− mice (means ± standard deviations of 3 experiments). (**H**) Malonaldehyde concentration in blood of control and Top1mt−/− mice (means ± standard deviations of 3 experiments). (**I**) Representative Western blotting experiment showing FASN (Fatty Acid Synthase) expression in liver from control and Top1mt−/− mice.

Analyses of O_2_ consumption rates (OCR) in Top1mt−/− and WT cells showed an increase in Top1mt−/− cells (Fig. 4A). Because O_2_ can be used for cellular fatty acid oxidation, a process that breaks fatty acids to release energy, and which involves mitochondria [35], we next measured fatty acid oxidation in Top1mt−/− cells. Cells were treated with C75, a fatty acid synthase (FASN) inhibitor [36], and fatty acid-dependent oxygen consumption rate was measured. Fatty acid oxidation (FAO) dependent-oxygen consumption rate (OCR) was about 2-fold decreased after C75 treatment in WT cells, whereas the decrease was about 3.3 fold in Top1mt−/− cells (Fig. 4B). We also observed that C75 treatment did not affect the extra cellular acidification rate (Fig. S1). Thus, our results are consistent with the hypothesis that Top1mt−/− cells are using O_2_ to execute fatty acid oxidation and to generate energy. FASN (Fatty Acid Synthase) was also increased about 2-fold in Top1mt−/− cells (Fig. 4C), suggesting that fatty acid synthesis (enhanced FASN) compensates lipid degradation by fatty acid oxidation.

To further investigate mitochondrial dysfunctions in Top1mt−/− cells, we checked glutathione production [37]. Glutathione production was elevated in Top1mt−/− cells (Fig. 4D), highlighting the fact that Top1mt−/− cells produce more ROS than WT cells. Because excess ROS creates cellular damage and lipid peroxidation, we also measured malonaldehyde (MDA), the end products of lipid peroxidation, in Top1mt −/− and WT cells. MDA quantity was increased in Top1mt−/− cells (Fig. 4E), suggesting that ROS in Top1mt−/− cells lead to high lipid peroxidation.

We next checked for the presence of oxidative damage in mouse liver using the anti-HNE marker by immunofluorescence. HNE (4-Hydroxy-2-nonenal) is a major product of endogenous lipid peroxidation and is a marker of oxidative stress [38]. Livers from Top1mt−/− mice showed increased HNE staining (Fig. 4F), suggesting oxidative stress in mice lacking Top1mt. The oxidative stress in Top1mt−/− liver is accompanied by an augmentation of the antioxidant glutathione (GSH) in Top1mt−/− mouse blood (Fig. 4G). As for the Top1mt−/− cells, oxidative damage was associated with lipid peroxidation in Top1mt−/− mice: the malonaldehyde content was almost 2-fold elevated in Top1mt−/− mice blood (Fig. 4H) and was associated with FASN protein overexpression (Fig. 4I).

### Lack of Top1mt Activates the DNA Damage Response (DDR) Pathways

**Figure 5:**
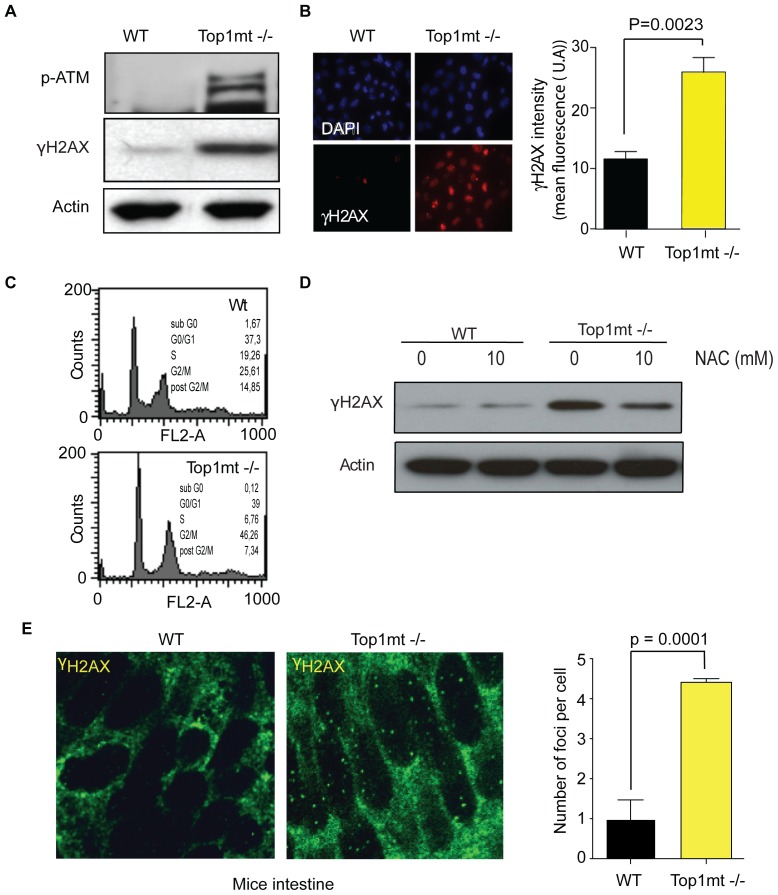
ATM-dependent DNA damage response pathway activation in Top1mt−/− cells. (**A**) γH2AX, pS1981-ATM and actin protein expression detected by Western blotting in WT ant Top1mt−/− cells. (**B**) γH2AX visualization by immunofluorescence and relative quantification in WT and Top1mt−/− cells. (**C**) Cell cycle analysis of WT and Top1mt−/− cells. (**D**) Detection of γH2AX by Western blotting after ROS inhibition with 10 mM of NAC for 3 h in WT and Top1mt−/− cells. (**F**) Immunostaining of intestine sections with γH2AX antibody in control and Top1mt−/− mice livers.

ROS are known to damage DNA and activate the DDR pathways mediated by ATM and γH2AX [39], [40]. Activation of ATM requires its autophosphorylation on serine 1981 [41], [42]. Western blot analysis with phospho-ATM (ser-1981) antibody revealed the presence of activated ATM (pS1981-ATM) in Top1mt−/− cells (Fig. 5A). Consistent with the presence of activated ATM in Top1mt−/− cells, histone γH2AX, a substrate of activated ATM and a marker of DNA double-strand breaks [43] was also elevated in Top1mt−/− cells (Fig. 5A and 5B). The induction of the DDR pathways in Top1mt−/− cells is associated with a slight reduction of S phase and an increased fraction of cells in G2/M phase of the cell cycle (Fig. 5C). Treatment of Top1mt−/− cells with the ROS inhibitor N-acetyl cystein (NAC) reduced γH2AX level (Fig. 5D), indicating that ROS induction participates in the endogenous activation of the DDR pathway in Top1mt−/− cells. We also looked for γH2AX activation in murine intestinal crypts and found increased γH2AX staining in Top1mt-deficient cells, consistent with the activation of the DDR pathways and ROS production in those cells (Fig. 5E).

### Lack of Top1mt Triggers Autophagy

**Figure 6:**
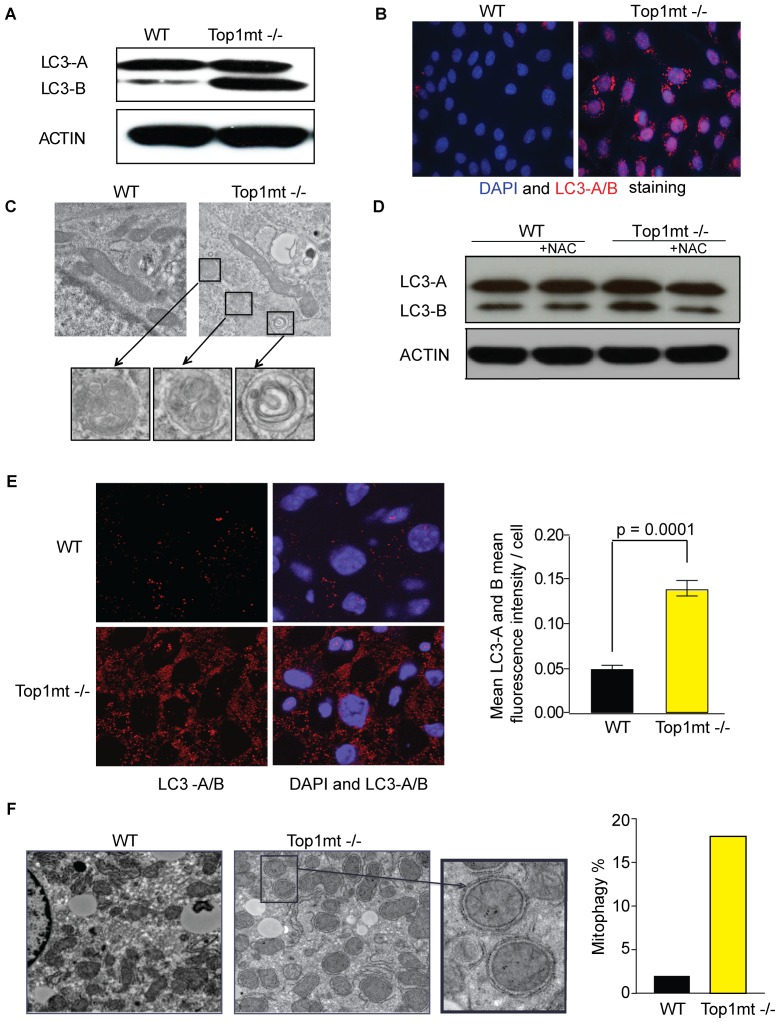
Enhanced autophagy in Top1mt−/− cells and mice liver. (**A**) Expression of LC3-A/B (microtubule-associated protein1 light chain 3) by Western blotting in WT and Top1mt−/− cells. (**B**) LC3-A/B and DAPI staining by immunofluorescence in WT and Top1mt−/− cells (**C**) Transmission electronic microscopy showing autophagic vesicules in WT and Top1mt−/− cells. (**D**) Expression of LC3-A/B by Western blotting in WT and Top1mt−/− cells in presence or absence of 10 mM NAC antioxidant. (**E**) LC3-A/B and DAPI staining by immunofluorescence in Top1mt−/− mice liver and relative quantification. (**F**) Transmission electronic microscopy showing mitophagy in control and Top1mt−/− mouse liver. Histogram corresponding to the proportion of mitochondria totally surrounded by cytoplasmic membrane (autophagosome) related to the total number of mitochondria.

Autophagy is a cellular recycling process responsible for the turnover of unnecessary or dysfunctional organelles and cytoplasmic proteins resulting from cellular stress [44]. Because ROS activate autophagy [45], [46] and Top1mt−/− cells have elevated ROS production (see above), we tested autophagy induction in Top1mt−/− cells using the autophagy marker Light Chain 3 (LC3). During autophagy, LC3-A is converted to LC3-B through lipidation by an ubiquitin-like system that allows LC3 to become associated with autophagic vesicles [47], [48]. The presence of LC3 in autophagosomes and the conversion of LC3 to the lower migrating form LC3-B have been used as indicators of autophagy. Figure 6 shows an increase of the LC3-B form in Top1mt−/− cells (panel A) and an increase of LC3-A/B staining by immunofluorescence in Top1mt−/− cells (panel B). Also, electronic microscopy pictures showed autophagic vesicles characterized by the presence of double membranes and engulfed cytosolic content in Top1mt −/− cells (Fig. 6C).

To determine whether autophagy in Top1mt−/− cells was related to increased ROS, we tested whether autophagy would be affected by NAC. Figure 6D shows that treatment of Top1mt−/− cells with NAC reduces LC3-B levels, indicating that increased ROS production in Top1mt−/− cells is involved in autophagy induction.

Finally we investigated autophagy in Top1−/− mice. Immunofluorescence experiments done with LC3 antibody showed a 2-fold increase in Top1mt−/− mouse liver (Fig. 5E). Electron microscopy of Top1mt−/− livers (Fig. 6F) also showed autophagosomes with engulfed mitochondria, which is commonly referred to as mitophagy [49], a selective autophagy of mitochondria. Taken together our results demonstrate elevated autophagy in Top1mt−/− cells, and mitophagy in the liver of Top1mt−/− mice.

## Discussion

We present the first evidence that Top1mt deficiency produces mitochondrial dysfunctions. In MEF cells, we observed a marked increase in ROS production, calcium signaling and hyperpolarization of mitochondrial membranes. Mitochondria hyperfusion in Top1mt −/− cells probably reflects a stress response to ROS overproduction. Activation of the ATM-dependent DNA damage response (DDR) pathway in Top1mt deficient mice tissues and MEFs is also consistent with the presence of elevated ROS as a result of Top1mt inactivation. Moreover, the endogenous DDR in Top1mt −/− cells visualized by histone γH2AX activation was significantly reduced after ROS quenching by NAC treatment, which is consistent with the possibility that ROS-dependent mitochondrial production elicits DNA damage in Top1mt −/− cells.

Top1mt-deficient cells are also resistant to the electron transport chain inhibitors rotenone and oligomycin A, which indicates that Top1mt-deficient cells utilize alternative pathways for cellular respiration and energy production. Mitochondrial respiratory chain defects due to the lack of Top1mt may be responsible for the increased ROS levels and for the compensatory increase of glutathione, an antioxidant protection mechanism, in Top1mt−/− cells and mouse blood.

Because cells get their energy through the OXPHOS mitochondrial oxygen-dependent pathway and through the oxygen-independent glycolysis pathway, decreased ATP levels in Top1mt−/− cells may be compensated by increased aerobic glycolysis for ATP production. Indeed, increased glucose uptake and lactate production associated with overexpression of the glucose transporter Glut1 and HIF-α was found in Top1mt-deficient cells indicative of enhanced glycolysis. The switch from OXPHOS to glycolysis is equivalent to the Warburg effect, which is a feature of cancer cells [12]. Consistent with the inefficiency of Top1mt cells at using the respiratory chain to produce ATP, oxygen consumption is elevated in those cells. This increased oxygen consumption in Top1mt −/− cells can also facilitate fatty acid oxidation, an aerobic process that generates ATP. In Top1mt−/− cells, inhibition of FA synthesis has a stronger effect on FAO inhibition compared to WT, suggesting that Top1mt−/− cells rely in part on lipids for oxygen consumption. We cannot exclude that Top1mt−/− cells use more oxygen for their metabolism because of an increase in mitochondrial mass.

Lipid peroxidation occurs in both MEFs and mice lacking Top1mt as shown by the expression of malonaldehyde. Moreover, overexpression of FASN suggests that lipid biogenesis may compensate for degradation of lipids by lipid peroxidation. Presence of mitophagy in Top1mt−/− cells, and autophagy in Top1mt−/− cells, are signs of mitochondrial and/or cellular dysfunctions. In Top1mt−/− liver, mitophagy could regulate mitochondrial number to match metabolic demand, removing damaged mitochondria, and promoting cell survival. Thus, upregulation of autophagy in Top1mt-deficient cells and liver may be a protective mechanism to eliminate damaged mitochondria, which otherwise would produce higher ROS levels and unsustainable genotoxic stress [50]. ROS accumulation in Top1mt−/− cells contributes to the autophagy phenotype since ROS inhibition by NAC significantly reduces autophagy. However, ROS production can also induce cell survival by engaging a mitochondria-nuclear communication referred to as the retrograde response [16], [21]. This process can upregulate nuclear-encoded mitochondrial genes involved in mitochondrial biogenesis, such as PGC1-α, the master regulator of mitochondrial biogenesis, TFAM, a regulator of mitochondrial replication and transcription, NRF-1, transcription factor implicated in the control of nuclear genes required for respiration, heme biosynthesis, mitochondrial DNA transcription and replication, and the mitochondrial polymerase POLG. We also found an increase of c-myc transcription and protein level, which is consistent to the notion that c-myc regulates mitochondrial biogenesis [17]. Moreover, transcript levels of the three mitochondrial cytochrome c oxidase, COX1; 2; 3 and transcript levels of ND2; 4; 5, involved in the complex I, are more elevated in Top1mt−/− cells.

SLP-2 protein, which is involved in mitochondrial hyperfusion [26], is upregulated in Top1mt −/− cells. Recent findings suggest that upregulation of SLP-2 is associated with increased expression of mitochondrially-targeted genes and mitochondrial mass [51], which is the case of Top1mt−/− cells. The mitochondrial hyperfusion we observed in Top1mt−/− cells could function as a cell survival mechanism, as hyperfusion has been involved in mitochondrial resistance to autophagy and apoptosis [52], [53]. Thus, SLP-2 could act as a coordinator of cell survival pathways in response to the enhanced autophagy in Top1mt deficient cells.

Taken together, our study demonstrates dysfunctional mitochondria in Top1mt knockout cells and mice associated with oxidative stress, engagement of the retrograde response, increased glycolysis, and autophagy. Those homeostatic changes are likely to contribute to the survival of Top1mt−/− cells and mice despite the presence of altered mitochondria.

## Materials and Methods

### Generation of the Top1mt −/− Mouse Embryonic Fibroblasts (MEF) Cell Line

The Top1mt−/− MEF cells were generated from Top1mt−/− mice, which were constructed by deletion of the last two exons of Top1mt gene [5].

### Cell Culture

MEF cells were isolated from wild type or Top1mt −/− mice by mechanical disaggregation and cells were grown in DMEM with l-glutamine, supplemented with 15% fetal calf serum. For glucose deprivation, cells were cultivated in glucose-free DMEM (Invitrogen, Cat. No 11966-025) supplemented with 15% of fetal calf serum, 10 mM galactose, 2 mM glutamine, 5 mM HEPES and 1 mM sodium pyruvate.

### Reagents

N-acetyl cystein, rotenone, oligomycin A and C75 were purchased from Sigma-Aldrich (St.Louis, MO). TMRM (tetramethylrhodamine methyl ester), Mitotracker red, Calcium Green™-1dye, CM-H_2_DCFDA were obtained from Molecular Probes CA, USA.

### Real Time PCR

Total RNA was isolated from 1×10^6^ cells using RNeasy® Mini Kit (Qiagen, Valencia, CA). An aliquot of 1 µg RNA was reverse transcribed using a reverse transcription kit (Promega). Real-time PCR was performed with The SYBR® Green PCR Master Mix (Applied Biosystems, Foster city, CA) on the ABI 7900 thermocycler (Applied). Reaction mixtures contained 5 µl of 2× Quantitect SYBR-Green PCR Master Mix, 2 µl of reverse-transcriptase-generated cDNA diluted by 100 in a final volume of 10 µl containing primers (IDT) at 125 nM. Relative gene expression was expressed as a ratio of the expression level of the gene of interest to that of β2 microglobulin (β2m) RNA, with values in MEF WT cells defined as 100%.

The sequences of the primers are listed in table S1.

### Mitochondrial Membrane Potential (Δψ_m_) and Reactive Oxygen Species (ROS) Production

Determination of ΔΨm was performed with Tetramethylrodhamine methyl ester (TMRM, Invitrogen). TMRM is a positively charged, colorless dye that enters mitochondria in a membrane potential-dependent manner. Once in mitochondria, this dye emits bright red-orange fluorescence that can be quantified by flow cytometry. Briefly, WT and Top1mt−/− MEFs were harvested, washed, and resuspended in HBSS buffer. Cells were then loaded with 200 nM TMRM for 30 min at 37°C and fluorescence was measured by FACScan flow cytometer (BD Biosciences) using the CellQuest software (BD Biosciences). In each analysis, 10000 events were recorded.

ROS production in cells was measured by CM-H_2_DCFDA (Invitrogen, Carlsbad, CA). CM-H_2_DCFDA is a cell-permeant indicator for ROS that is non-fluorescent until oxidation within the cell. Cells were seeded at 2×10^6^ cells/ml in 6 well plates. The next day cells at density of 1×10^6^ cells/ml were trypsinized and washed in PBS. The cells were resuspended with 1 ml of pre-warmed PBS to 37°C with 10 µM of CM-H_2_DCFDA dye and incubated for 30 minutes at 37°C in the dark. Cells were then washed in PBS, resuspended in a total volume of 500 µl, and immediately analyzed by flow cytometry. Cells were analyzed with a FACScan flow cytometer (BD Biosciences) using the CellQuest software (BD Biosciences).

### Immunoblotting

Cells were lysed at 4°C in buffer containing 1% SDS, 10 mM Tris-HCl pH 7.4, supplemented with protease inhibitors (Roche Applied Science, Indianapolis, IN) and phosphatase inhibitors (Sigma Chemical Co., St. Louis, MO). Viscosity of the samples was reduced by brief sonication. 40 µg of protein (Bio-Rad Protein Assay) were boiled for 5 min in Tris-glycine-SDS sample buffer (Invitrogen) and heated at 70°C for 10 min, then separated by SDS-polyacrylamide gel electrophoresis, and transferred onto nitrocellulose membranes (Bio-Rad, Hercules, CA). After blocking nonspecific binding sites for 1 h with 5% milk in TPBS (phosphate-buffered saline, Tween20 0.1%), membranes were incubated overnight with primary antibody. After three washes in TPBS, the membrane was incubated with horseradish peroxidase-conjugated goat anti-rabbit (1∶10,000 dilution), goat anti-rabbit (10,000 dilution), or anti-mouse (1∶5,000 dilution) antibody (Amersham Biosciences, Piscataway, NJ) for 1 h and then washed three times in TPBS. Immunoblots were revealed using enhanced chemiluminescence detection kit (Pierce) by autoradiography.

The primary antibodies used were: anti-γH2AX (NB100-384, Novus Biologicals, Littleton, CO), anti-TFAM (sc-23588; Santa Cruz Biotechnology, Santa Cruz, CA), anti-PGC-1 (sc-13067; Santa Cruz Biotechnology), anti-POLG (ab128899, Abcam), anti HIF-1α (sc-10790; Santa Cruz Biotechnology), anti-FASN (3189; Cell signaling Inc., Danvers, MA), anti-Glut1 (#GTX62480, GeneTex Inc, Irvine, CA), anti-LC3-A/B (L10382, Invitrogen), anti-SLP2 (sc-98709; Santa Cruz Biotechnology), anti-C-myc (sc- 47694, Santa Cruz), anti-ATM (Ser 1981) (4526, Cell Signaling) and anti-Actin (A3853, Sigma, St. Louis, MO). Top1mt antibody was generated by immunizing mice with recombinant human Top1mt.

### Glucose Uptake Assay

Glucose uptake was measured as previously described [54] using a non-metabolized fluorescent D-glucose analog 2-[N-(7-nitrobenz-2-oxa-1,3-diazol-4-yl) amino]-2-deoxy-d-glucose (2-NBDG) (Cayman Chemicals, Ann Arbor, MI) with the following modifications. Ten thousand cells were plated in black-well 96-well plates. After treatment, cells were incubated in PBS containing 1 g/L glucose in presence of 2-NBDG (20 µM). After 20 minutes incubation and several washes, cells were incubated in DMEM without phenol red and the uptake of 2-NBDG was measured by spectrophotometry.

### Measurements of Extracellular Acidification and Oxygen Consumption Rate

The XF96 Extracellular Flux Analyzer from Seahorse Bioscience (Chicopee, MA) was used to measure cellular respiration (O_2_ consumption, OCR) and extracellular acidification rate (ECAR). Cells were cultured in custom XF96 microplates. All measurements were performed following manufacturer’s instructions and protocols [55], and the observed rates were reported in pMol/min for OCR and mpH/min for ECAR. Briefly, cells were seeded at 10,000 cells per well in XF96 well culture microplates. Twenty four hours later, culture media was removed from the XF culture plates containing the treated and untreated cells. The wells were washed and incubated with assay medium (bicarbonate-free DMEM supplemented with glucose (4.5 g/L) and sodium pyruvate (2 mM) pH 7.4 at 37°C). For the fatty acid oxidation experiments, we used low-buffered KHB buffer (110 mM NaCl, 4.7 mM KCl, 2 mM MgSO_4_, 1.2 mM Na_2_HPO_4_, 2.5 mM glucose) adjusted at pH 7.4 at 37°C and supplemented with 0.5 mM carnitine, 100 nM insulin, 200 µM palmitate.

### Determination of Mitochondrial Mass

Mitochondrial mass was measured by Mitotracker Green FM (Molecular Probes) staining. Cells were trypsinized and resuspended in PBS with 1 µM Mitotracker Green FM. After incubation for 25 min at 37**°**C in the dark, cells were transferred immediately to a tube on ice for flow-cytometry analysis.

### Immunofluorescence Microscopy

Cells were washed with PBS, fixed with 2% formaldehyde in PBS for 20 min, washed with PBS, fixed and permeabilized with pre-chilled (−20°C) 70% ethanol for 20 min, washed with PBS, blocked with 8% bovine serum albumin (BSA) in PBS for 1 h, washed with PBS, incubated with the first antibody: anti-LC3-A/B (L10382, Invitrogen), 1/500 dilution; anti-γH2AX (NB100-384, Novus Biologicals), (1/500 dilution); anti HNE (393206, Calbiochem, San Diego, CA), (1∶500 dilution), in 1% BSA in PBS. After incubation for 2 h, slides were washed with PBS, incubated with the secondary antibody conjugated with Alexa Fluor 488 or 568 for 1 h at room temperature, washed with PBS, and mounted with Vectashield mounting medium with DAPI (4′, 6′-diamidino-2-phenylindole) to counterstain the DNA (Vector Laboratories, Burlingame, CA). Slides were examined using a laser scanning confocal microscope (Zeiss LSM510). Images were collected and processed using the Zeiss AIM software. Level of fluorescence intensity was realized with ImageJ software.

### Immunohistochemistry

Sections (5 µm thick) of frozen OCT-embedded mouse livers were fixed in 2% paraformaldehyde in PBS for 20 min and permeabilized in pre-chilled (−20°C) 70% ethanol. Sections from wild type mice and Top1mt −/− mice were treated as above.

### Immunohistochemistry on Paraffin Sections

Intestine tissues were fixed in 10% neutral buffered formalin. Five micron sections from the paraffin-embedded tissues were done. Heat antigen retrieval was performed for 25 min at 95°C in sodium citrate buffer (10 mM sodium citrate, 0.05% Tween20, pH 6) and then sections from wild type and Top1mt −/− mice were treated as above. Tissues were stained with anti-γH2AX (phospho Ser 135) (NB100-384, Novus).

### Cell Cycle Analysis

Cells were washed with PBS, fixed, and permeabilized with cold (−20°C) 70% ethanol overnight at 4°C. The next day, cell pellets were washed again with PBS, resuspended in PBS buffer containing 0.5 mg/ml RNase A, incubated at room temperature for 15 min, and put on ice 10 min prior the addition of 50 µg/ml propidium iodide. Cell cycle analysis was determined with a FACScan flow cytometer (Becton Dickinson) and quantified with CellQuest software (Becton Dickinson, Sparks, MD).

### Mitochondrial Hyperfusion

Mitochondrial morphology was determined by cell loading with 10 nM MitoTracker Red, a specific mitochondrial dye, for 30 min. Cells were rinsed and fixed for 30 min with 2% paraformaldehyde in PBS. Mitochondrial morphology was observed with confocal microscopy as described above.

### MTS Assay

Briefly, cells were plated at a density of 10^4^ cells/well in 96-well plates.

After overnight incubation, the plates were treated with various concentrations of drugs and incubated at 37°C for 72 h. Cell proliferation was assayed by adding 20 µl of 3-(4,5-dimethylthiazol-2-yl)-5-(3-carboxymethoxyphenyl)-2-(4-sulfophenyl)-2H-tetrazolium (MTS) (Promega) to each well. Plates were then incubated at 37°C for 1 h and read at 490 nm. Analysis was performed on triplicate wells, and the data presented is representative of three independent experiments.

### Glutathione Assay

Detection and quantification of glutathione (GSH) in cell lysates or mouse erythrocytes were performed using the luminescence-based assay GSH-Glo™ Glutathione Assay (Promega), and following the manufacturer’s protocol.

### ATP Determination

ATP level were determined using an ATP determination Kit (Molecular Probes) and following the manufacturer’s protocol.

### Lactate Production

Cellular lactate was measured under normoxia with a fluorescence-based lactate assay kit (MBL International Corp, Woburn, MA). Phenol red–free DMEM medium without FBS was added to a six-well plate of subconfluent cells. After incubation at 37°C, 1 µl of medium from each well was assessed with the lactate assay kit.

### Calcium Measurement

The cell permeant dye, Calcium Green™-1 (Invitrogen), was used to monitor the intracellular calcium concentration. Cells were trypsinized and resuspended in PBS with 1 µM Calcium Green™-1. After incubation for 25 min at 37**°**C in the dark, cells were transferred immediately to a tube on ice for flow-cytometry analysis.

### Transmission Electron Microscopy

Samples were collected and fixed in 2.5% glutaraldehyde in 0.1 M cacodylate buffer (pH 7.4) for 2 h. After rinsing with cacodylate buffer, the samples were fixed in 1% osmium tetroxide for 1 h. Samples were then stained with 0.5% uranyl acetate, dehydrated in graded ethanol and propylene oxide, and infiltrated in equal volume of epoxy resin and propylene oxide overnight. Sample were embedded in epoxy resin and cured in 55°C oven for 48 h. The cured block was thin-sectioned and stained in uranyl acetate and lead citrate and examined by electron microscopy (Hitachi H700 microscope, Tokyo, Japan).

## Supporting Information

Figure S1 **Glycolysis is enhanced by inhibition of fatty acid synthase.** Extra-cellular acidification rate measured by seahorse in WT and Top1mt−/− cells after C75 treatment for 3 h.
(TIFF)

Table S1 **Primers sequences used in this study.**
(DOC)
