# Supplementary figures and images for: Mitochondrial Topoisomerase I is Critical for Mitochondrial Integrity and Cellular Energy Metabolism
Source: PLoS One. 2012 Jul 20;7(7):e41094. doi: 10.1371/journal.pone.0041094 (PMC3401127; doi:10.1371/journal.pone.0041094)

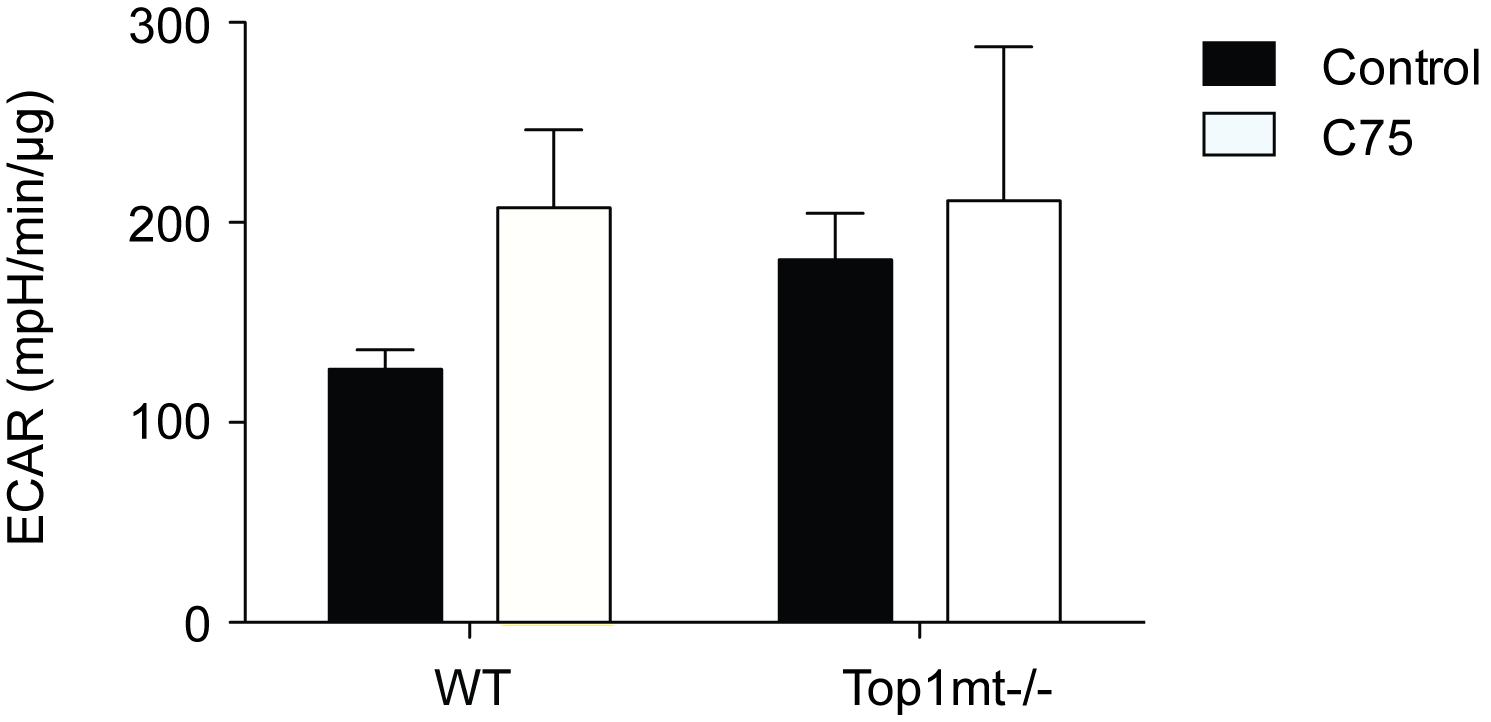

Supplement: Figure S1 — Glycolysis is enhanced by inhibition of fatty acid synthase. Extra-cellular acidification rate measured by seahorse in WT and Top1mt−/− cells after C75 treatment for 3 h. (TIFF) [file pone.0041094.s001.tiff]
