# Supplementary material for: Mitochondrial Topoisomerase I is Critical for Mitochondrial Integrity and Cellular Energy Metabolism
Source: PLoS One. 2012 Jul 20;7(7):e41094. doi: 10.1371/journal.pone.0041094 (PMC3401127; doi:10.1371/journal.pone.0041094)
Supplement: Table S1 — Primers sequences used in this study. (DOC) [file pone.0041094.s002.doc]

| gene | Forward primer | Reverse primer |
| --- | --- | --- |
| mouse β2m | 5’-TCTTTTTCAGTGGGGGTGAA-3’ | 5’-CTCACGTCATCCAGCAGAGA-3’ |
| mouse POLG | 5’-GCAGGATGGGCAGGAACA-3’ | 5’-GCATCCGGGAGTCCTGAA-3’ |
| mouse NRF-1 | 5’-CTGAGCCTGGGTCATTTTGT-3’ | 5’-CCACGTTGGATGAGTACACG-3’ |
| mouse PGC-1 | 5’-TGGTTTGCTGCATGGTTCTG-3’ | 5’-GGAGCCGTGACCACTGACA-3’ |
| mouse CytB | 5’-ATTCCTTCATGTCGGACGAG-3’ | 5’-ACTGAGAAGCCCCCTCAAAT-3’ |
| mouse mt-ND2 | 5’-CCATTCCACTTCTGATTACC -3’ | 5’-GTCATGTAAGAAGAATAAGTCC-3’ |
| mouse TFAM | 5’-CATTTATGTATCTGAAAGCTTCC-3’ | 5’-CTCTTCCCAAGACTTCATTTC-3’ |
| mouse Cox1 | 5’-TTTTCAGGCTTCACCCTAGATGA-3’ | 5’-CCTACGAATATGATGGCGAAGTG-3’ |
| mouse Cox2 | 5’-TGAAGACGTCCTCCACTCATGA-3’ | 5’-GCCTGGGATGGCATCAGTT-3’ |
| mouse Cox3 | 5’-GTTTGCCTACGACAACTAAAATTTC-3’ | 5’-TGCTGCGGCTTCAAATCC-3’ |
| human B2m | 5’-TCTTTTTCAGTGGGGGTGAA-3’ | 5’-CTCACGTCATCCAGCAGAGA-3’ |
| human TFAM | 5’-CAAGTATTATGCTGGCAGAAGTCC-3’ | 5’-AACAACGAAAATATGGTGCTGAGG-3’ |
| mouse c-myc | 5’-ACAGGACTCCCCAGGCTCCG-3’ | 5’-CGTGGCTGTCTGCGGGGTTT-3’ |
| mouse Top1mt | 5’-CCTTGACAAATGTGACTTCACG-5’ | 5’-GACCACATCCTCTGGCATGAC-3’ |
| mouse mt-ND4 | 5’-CCAGCCTAACACTTCTATG-3’ | 5’-GGCTAGCTATTAATATTAGTGGC-3’ |
| mouse mt-ND5 | 5’-CCTACTAATTGGATGATGGTAC -3’ | 5’-CGGTTATAGAGGATTGCTTG-3’ |
